# Lactonic Sophorolipids Increase Tumor Burden in Apc^min+/-^ Mice

**DOI:** 10.1371/journal.pone.0156845

**Published:** 2016-06-06

**Authors:** Breedge Callaghan, Helen Lydon, Sophie L. K. W. Roelants, Inge N. A. Van Bogaert, Roger Marchant, Ibrahim M. Banat, Christopher A. Mitchell

**Affiliations:** 1 Biomedical Sciences Research Institute, Centre for Molecular Biosciences, Ulster University, Coleraine, BT52 1SA, United Kingdom; 2 Centre for Industrial Biotechnology and Biocatalysis (InBio.be), Faculty of Bioscience Engineering, Ghent University, Coupure Links 653, 9000, Ghent, Belgium; University of Kentucky, UNITED STATES

## Abstract

Sophorolipids (SL) are amphiphilic biosurfactant molecules consisting of a disaccharide sophorose with one fatty acid at the C1 position and optional acetylation at the C6’and C6” positions. They exist in a closed ring lactonic (LSL) or open acidic (ASL) structure Sophorolipids are produced in crude mixtures in economically viable amounts by the yeast *Starmerella bombicola* and used in a variety of consumer products. Varying levels of anti- proliferative and anti-cancer activity of crude sophorolipid mixtures are described in a number of tumor cell lines *in vitro*. However, significant inter-study variation exists in the composition of sophorolipid species as well as other biologically active compounds in these mixtures, which makes interpretation of *in vitro* and *in vivo* studies difficult. We produced a 96% pure C18:1 lactonic sophorolipid that dose-dependently reduces the viability of colorectal cancer, as well as normal human colonic and lung cell lines *in vitro*. Oral administration of vehicle-only; or lactonic sophorolipids (50 mg/kg for 70 days), to Apc^min+/-^ mice resulted in an increase in the number (55.5 ± 3.3 *vs* 70.50 ± 7.8: p < 0.05) and size (modal size 2mm vs 4mm) of intestinal polyps. Lactonic administration resulted in a systematic effect via reduced hematocrit (49.5 ± 1.0 vs 28.2 ± 2.0 *vs*: p<0.03) and splenomegaly (0.56 ± 0.03g vs 0.71 ± 0.04g; p<0.01) confirming exacerbation of disease progression in this model.

## Introduction

Biosurfactants are produced by a variety of microorganisms as secondary metabolites, forming emulsions that reduce both interfacial and surface tension [1]. Due to their increased biodegradability, low toxicity and ability to exert an effect at extreme temperatures and pH levels [2], they prove versatile for a wide range of biomedical and industrial applications [3]. Currently, a range of microbial biosurfactants are used in cleaning supplies [4], pesticides [5], textiles [6] and cosmetics [7] while petroleum derived surfactants are still used in food products [8] and over the counter creams [9]. Microbial biosurfactants are a diverse group of surface- active compounds classified by their chemical structure, weight and microbial origin [10]. Some well-known glycolipid biosurfactants include sophorolipids (SL), mannosylerythritols, trehalolipids and rhamnolipids [11].

**Fig 1:**
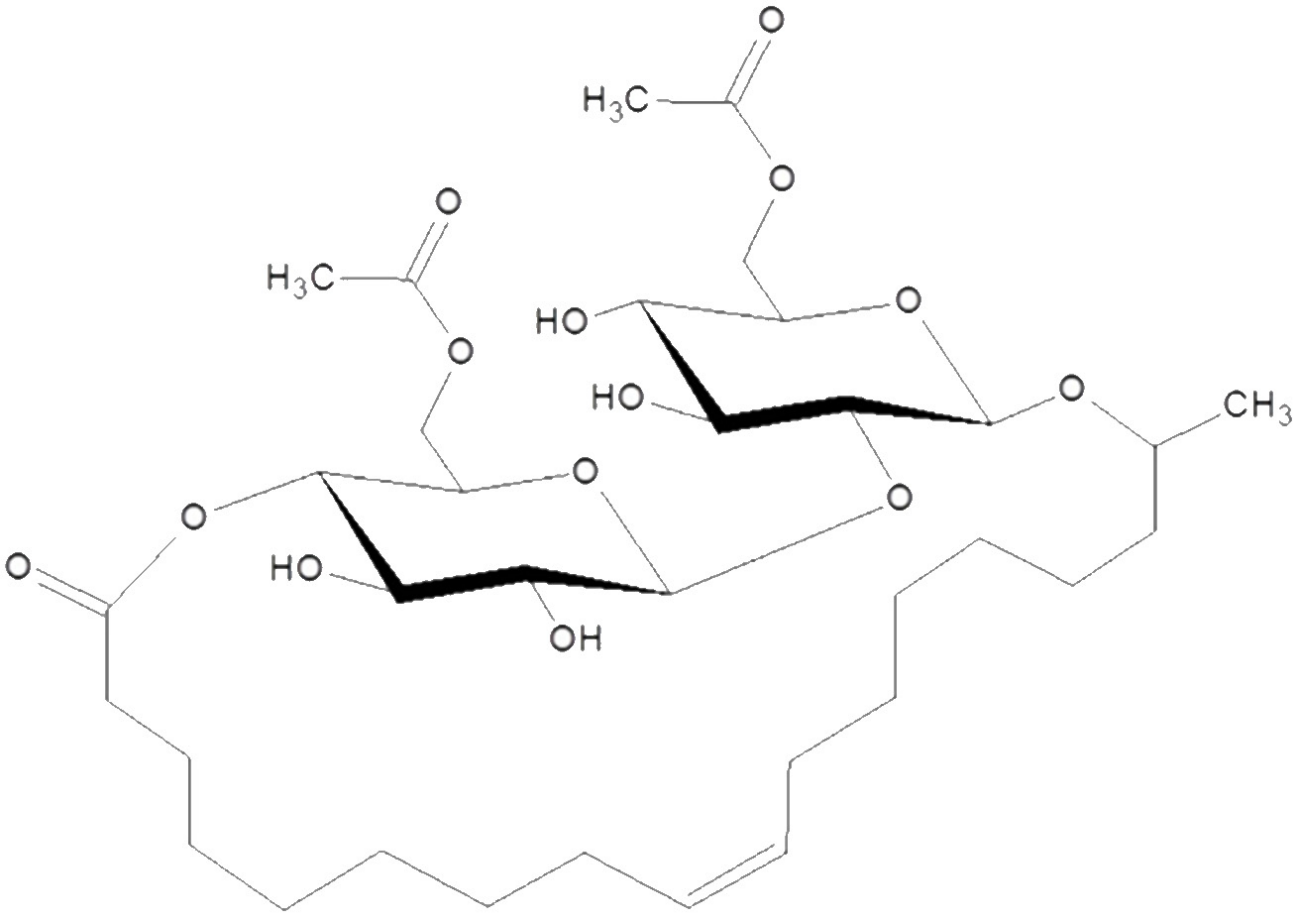
Lactonic Sophorolipid. The structure of C18:1 diacetylated LSL.

The SL species we describe in this study are produced by different types of yeast such as *Starmerella bombicola*, *Candida bastistaeic*, *C*. *floricola and C*. *apicola* [12]. In these organisms the SL species are composed of a hydrophobic fatty acid tail and a hydrophilic carbohydrate head composed of a disaccharide sophorose linked by a β-1, 2 bond which is optionally acetylated on the 6’ and/or 6” position. The structure of SLs is dependent on a terminal or sub-terminal hydroxylated fatty acid, which is linked β-glycosidically to the sophorose. The fatty acids’ carboxylic end can be free, forming the acidic structure [13] or can be esterified at the 4” position giving rise to the lactonic ring structure (Fig 1).

A wide range of bioactivities for SL have been documented; including antimicrobial activity *via* membrane destabilization and increased permeabilization [14] and anti-inflammatory effects through the reduction of cytokine release and initiation of a macrophage response [15]. Several studies indicate that LSL [16–18] show greater potentials as anti-tumor, anti-microbial, anti- fungal and spermicide agents, while ASL are more suited as moisturizing, solubilizing, cleaning and emulsifying agents. The purity and composition of SL used in bioassays is highly variable, with most studies not disclosing the molecular species or their relative abundances within the mixtures [19–21]. Additionally, SL analogs and their derivatives can reduce the efficacy of crude preparations [16, 19] and they are known to exhibit varying potencies and toxicities depending on their manufacturing methods [22]. LSL normally make up the highest proportion of crude preparations of SL (characteristically 70–85% [23]), with the remainder comprised of varying amounts of ASL and other derivatives; the aforementioned considerations underline the need to use purified and characterized (ASL or LSL) forms when assessing or comparing bioactivities. In order to minimize batch to batch variation and reduce specific congeners (2.5% ASL and <1% free fatty acids as a proportion of dry weight) we used a tightly-controlled batch fermentation method in order to produce a stock of highly pure and well characterized LSL that was used in all our *in vitro* and *in vivo* studies.

The *in vitro* activities of SL has been reviewed [12], with enriched preparations showing potent cytotoxicity against human liver (HT402), lung (A549) and leukemic (HL60 & K562) cells [24]. Cruder preparations of SL are reported to show dose-dependent, anti-proliferative and pro- apoptotic activity against pancreatic cancer (H7402, HPAC) [19] and esophageal cancer (KYSE450, KYSE109) cell lines [17]. Conclusions on the specificity of such diverse preparations of SL to transformed cells is complicated by the inappropriate use of controls—many studies lack the use of appropriate primary or non-transformed cells such as non-adherent peripheral blood mononuclear cells (PBMCs) [25].

Despite a number of studies showing that SL preparations have anti-proliferative effects on tumor cell lines, to our knowledge the anti-tumor effects of these compounds *in vivo* has not been reported. However, SL mixtures have been shown to reduce mortality and regulate nitric oxide production in a rat model of peritoneal sepsis [14], as well as reducing IgE production [26] following nebulizer administration in a mouse model of asthma [27]. These pre-clinical studies are consistent with the proposal that parenteral administration of relatively low doses of SL is safe and non-toxic in-*vivo*.

We hypothesized that purified forms of LSL would specifically inhibit colorectal tumor cell growth both *in vitro* and *in vivo*. Therefore, we purified and characterized a LSL preparation produced by *Starmerella bombicola* and assessed its’ effects on five unrelated colorectal cancer cell lines: HT29, HT115, HCT116, Caco-2 and LS180, in addition to two non-transformed lines: normal human colonic epithelium CCD-841 CoN and lung fibroblast MRC5. In addition, we administered 50mg/kg of LSL orally for 70 days to Apc^min+/-^ mice (a well-established model of colorectal neoplasia) [28, 29], to determine its’ ability to inhibit tumor growth *in vivo*.

## Materials and Methods

### Sophorolipid production and purification

A lactonesterase overexpressing strain of *Starmerella bombicola* (oe *sble*) as described by Roelants *et al*. [30] was used for the production of 96% pure lactonic diacetylated SL. HPLC- ELSD and LC-MS analysis was performed as described by Roelants *et al*. [30].

### Cell culture

The colorectal cancer cell lines HT29 (ATCC^®^ HTB-38), HT115 (ECACC-cultures 85061104) HCT116 (ATCC^®^ CCL-247), LS180 (ATCC^®^ CL-187), Caco-2 (ATCC^®^ HTB-37), normal colonic epithelium CCD-841-CoN (ATCC^®^ CRL-1790) and lung fibroblasts MRC5 (ATCC^®^ CCL-171) were maintained in DMEM media supplemented with 10% fetal bovine serum (Gibco Invitrogen; Paisley, UK). All cultures were maintained at 37°C and at 5% CO2.

### MTT assay

A total of 5x10^4^ cells per well were seeded (96 well plate: Nunc Thermos scientific, UK) and allowed to attach overnight before being serum starved for 24h. Various concentrations of LSL (0.001 μg/ml– 100 μg/ml) were added to the cultures and incubated for another 24h. Subsequently, 10μl of a 25mg/ml solution of MTT (3-(4, 5-dimethylthiazol-2-yl) -2, 5- diphenyltetrazolium bromide; (Sigma-Aldrich Company Ltd, Dorset, UK) was added to each well and the plate was further incubated for 1h at 37°C. The formazan crystals were solubilized with 100μl of DMSO (Sigma-Aldrich Company Ltd, Dorset, UK) and the absorbance at 570nm was read on a spectrophotometer plate-reader (BMG-LABTECH, Omega, Aylesbury, Bucks UK). Each experiment was repeated 3 times with 6 internal repeats per group.

### Acridine orange/ethidium bromide staining and quantitation

To determine the number of necrotic or apoptotic cells induced by addition of LSL, cells were stained *in situ* with 10mg/ml acridine orange (Sigma-Aldrich Company Ltd, Dorset, UK) and 1mg/ml ethidium bromide (Sigma-Aldrich Company Ltd, Dorset, UK) and morphological changes were assessed by fluorescence microscopy [31]. For assessment of apoptosis, a total of 3x10^4^ cells were seeded onto a 10mm coverslip (Agar Scientific; Stansted, Essex, UK) and incubated overnight to form a confluent monolayer. Following serum starvation for 24h, LSL (20μg/ml or 70μg/ml) or 5μM of etoposide (control) (Sigma-Aldrich Company Ltd, Dorset, UK) was added and the cells incubated for a further 24h. To determine the number of live cells remaining on the coverslip the samples were washed three times with ice-cold phosphate buffered saline (PBS; pH7.4, Oxoid: UK) 3 times, followed by incubation with a solution of 10μl of 1:1 acridine orange/ethidium bromide for 5 minutes and then the cells were washed 3x with ice-cold PBS and subsequent imaging with a Zeiss florescence microscope (Axio Scope 1, Zeiss, Germany) at a range of objective magnifications. The operator was blinded to the experimental groups and random fields were selected (40X objective). A total of 300 attached cells per coverslip were morphologically identified and counted as being either necrotic (red/orange nuclei), apoptotic (green condensed or fragmented nuclei) green or live (green non-condensed ovoid or rounded nuclei).

### Animal model

All animal procedures were approved by the animal care and ethics committee (Ulster University) and national (UK Home Office) ethical guidelines, and also carried out in accordance with both local animal care committee (Ulster University) and national (UK Home Office) guidelines by licensed personnel [32]. Apc^min+/-^ male and wild type (*wt*) female mice were housed together for breeding purposes and subjected to a 12/12 light cycle. Food and water were available *ad libitum* and weighed on a weekly basis to evaluate consumption. Animal husbandry was carried out bi-weekly. Mice were monitored on a daily basis looking at grooming, behavior, activity levels, food and water in-take and general well-being. Mice deemed un-well were immediately removed from the study and euthanized. Body weights were monitored bi-weekly. A cut-off point of 10% loss of body weight was applied and mice reaching this threshold were euthanized immediately by CO2.

### Genotyping

Ear punch samples were obtained from the 21 day old progeny of Apc^min+/-^/*wt* crosses for the purpose of genotyping, using primers specific for the APC mutation. DNA was isolated from ear samples by first solubilizing them in an alkaline lysis reagent (25mM NaOH, 0.2MmM disodium EDTA; (Sigma-Aldrich Company Ltd, Dorset, UK) at 95°C for 40 minutes, allowing them to cool and neutralizing in Tris-HCl (40mM; (Sigma-Aldrich Company Ltd, Dorset, UK).

Digested samples were mixed with PCR master mix (Qiagen Company Ltd, Manchester, UK), *taq* (Qiagen Company Ltd, Manchester UK), nuclease free water and Apc^min+^ specific primers (100μm each) (Forward: TCT CGT TCT GAG AAA GAC AGA AGC T, Reverse: TGA TAC TTC TTC CAA AGC TTT GGC TAT; Invitrogen Company Ltd, Paisley, UK). Samples were placed in a thermocycler (Techne TC-5000 Gradient Thermocycler, Hanwell, London UK) and a PCR reaction performed under the following conditions: 94°C, 2min; (94°C, 1min; 60°C, 1min; 72°C, 1min) for 30 cycles followed by 72°C for 2 minutes. PCR products were subjected to *Hind*III digestion (Invitrogen, Paisley, UK) for 1h at 37°C followed by a 20 minute denaturing step at 65°C. Digests were run on a 4% agarose/TBE-buffered gel (Sigma-Aldrich, Dorset, UK) for 40 minutes. The presence of a single band at 111bp indicated a *wt* mouse, while an additional band at 123bp indicated a heterozygous Apc^min+/-^ mouse.

### Sophorolipid dosing

At five weeks of age, equal numbers of *wt* and Apc^min+/-^ males and females were placed into experimental groups. Mice were treated orally (*via* a sterile p20 pipette tip) every other day with either vehicle-only or a solution containing 50mg/kg (body weight) of LSL suspended in 0.1% ethanol/ 10% sucrose for 70 days.

### Tissue collection and assessment

Mice were euthanized with an overdose of general anesthetic, blood immediately collected by cardiac puncture into EDTA tubes (Aquilant Scientific, Down, NI) and hematocrit determined (Cole-parmer, Trickenham,UK). Intestinal tract, colon spleen, heart, liver, kidneys and lungs were carefully removed, weighed and then fixed in 10% buffered formal saline (pH7.4). The intestinal tracts were divided into 3 sections according to the description of Casteleyn *et al*. (2010) [33]. After identification of the specific intestinal regions, samples were bisected longitudinally and the number of polyps was recorded as well as their diameters measured with calipers. The specimens were then cut into ~2cm strips and placed in cassettes prior to standard tissue processing and wax embedding. To assess qualitative histopathological changes in the intestines and spleen, tissues were cut into 5μm sections using a microtome (Shandon; Cheshire, UK) placed on glass slides, cleared with xylene, dehydrated in descending grades of ethanol, stained with Mayer’s haematoxylin and eosin stain (Sigma-Aldrich, Dorset, UK) and examined with a Zeiss light microscope (Axio Scope 1, Zeiss, Germany) at a range of objective magnifications.

### Statistical analysis

Statistical analysis of *in vitro* data was determined using either one-way ANOVA or student’s t- test using GraphPad Prism (GraphPad software, San Diego, USA). All comparisons between *in vivo* groups were assessed using a students’ t-test. A value of p <0.05 was defined statistically significant.

## Results

### Production and purification of LSL

**Fig 2:**
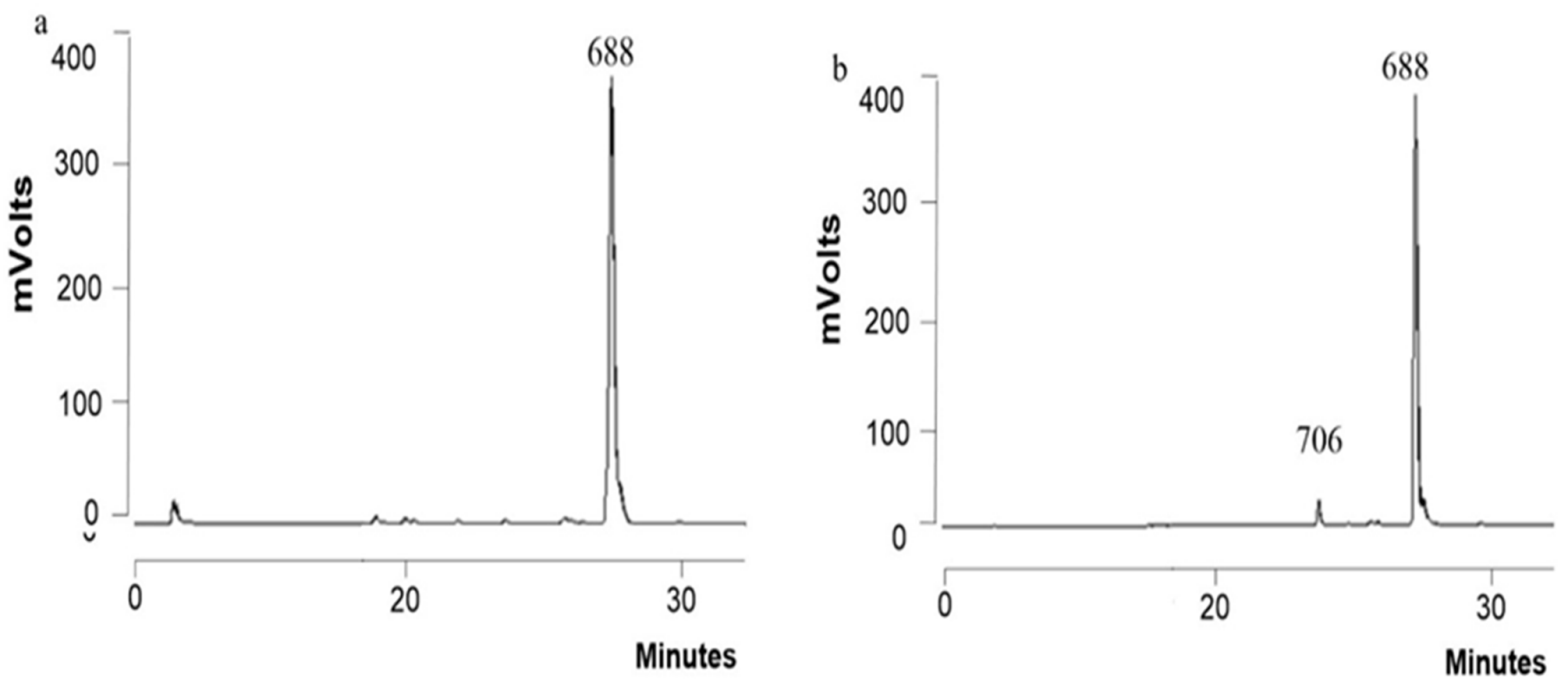
Purification of LSL produced by S. bombicola. HPLC-ELSD and LC-MS analysis of the LSL precipitate from the bioreactor. (a) The MW 688 g/mol corresponds to diacetyl C18:1 LSL. (b) HPLC-ELSD analysis following purification of the LSL precipitate. The MW 706 g/mol = diacetyl C18:1 ASL, which is the result of minor hydrolysis of the MW 688 compound.

To produce the C18:1 lactonic diacetylated SL used for our *in vitro* and *in vivo* studies (Fig 1), we employed the *S*. *bombicola* oe *sble* strain and a bioreactor experiment similar to one previously described [30]; however, instead of rapeseed oil, oleic acid was used as the hydrophobic carbon source. This resulted in a very uniform SL product (Fig 2a) containing 99% SL (97.3% C18:1 SL [Mw = 688], 1.3% C18:2 SL and 0.4% C18:0 SL).

The purification process was previously described [30] and consists of several washing steps with demineralized water of the spontaneously precipitated LSL product from the bioreactor. The washing steps remove hydrophilic impurities such as salts, sugars and proteins from the water-insoluble LSL. Hydrophobic impurities did not have to be removed, because the feeding rate of oleic acid was adjusted to its consumption rate, to avoid accumulation and the presence of an excess of substrate at the end of the fermentation. This is a large advantage, as solvent extractions to remove oil/fatty acids can be avoided. A final crystallization step of the LSL at 4°C, followed by lyophilisation gave rise to a dry and stable white powder. The final composition of the sample was analyzed using HPLC-ELSD and determination of possible congeners (e.g. salts, sugars proteins and oleic acid) was performed. The final purity of the sample was >99.5% SL and the composition in terms of SL was determined to be 96% diacetylated C18:1 LSL (Mw = 688; Fig 2b), 3.8% diacetylated C18:1 ASL (Mw = 706; Fig 2b) and minor impurities consisting of 0.04% free fatty acids/oil, 0.001% glucose, 0.004% glycerol and total nitrogen of 0.14%. The ASL was generated by hydrolysis of the LSL in the first step of the purification, which consisted of heating (65°C) of the culture broth, to melt and subsequently precipitate the SL product. This process was later optimized in order to avoid this unwanted hydrolysis [30].

### LSL have a differential effect on colorectal cell viability

**Fig 3:**
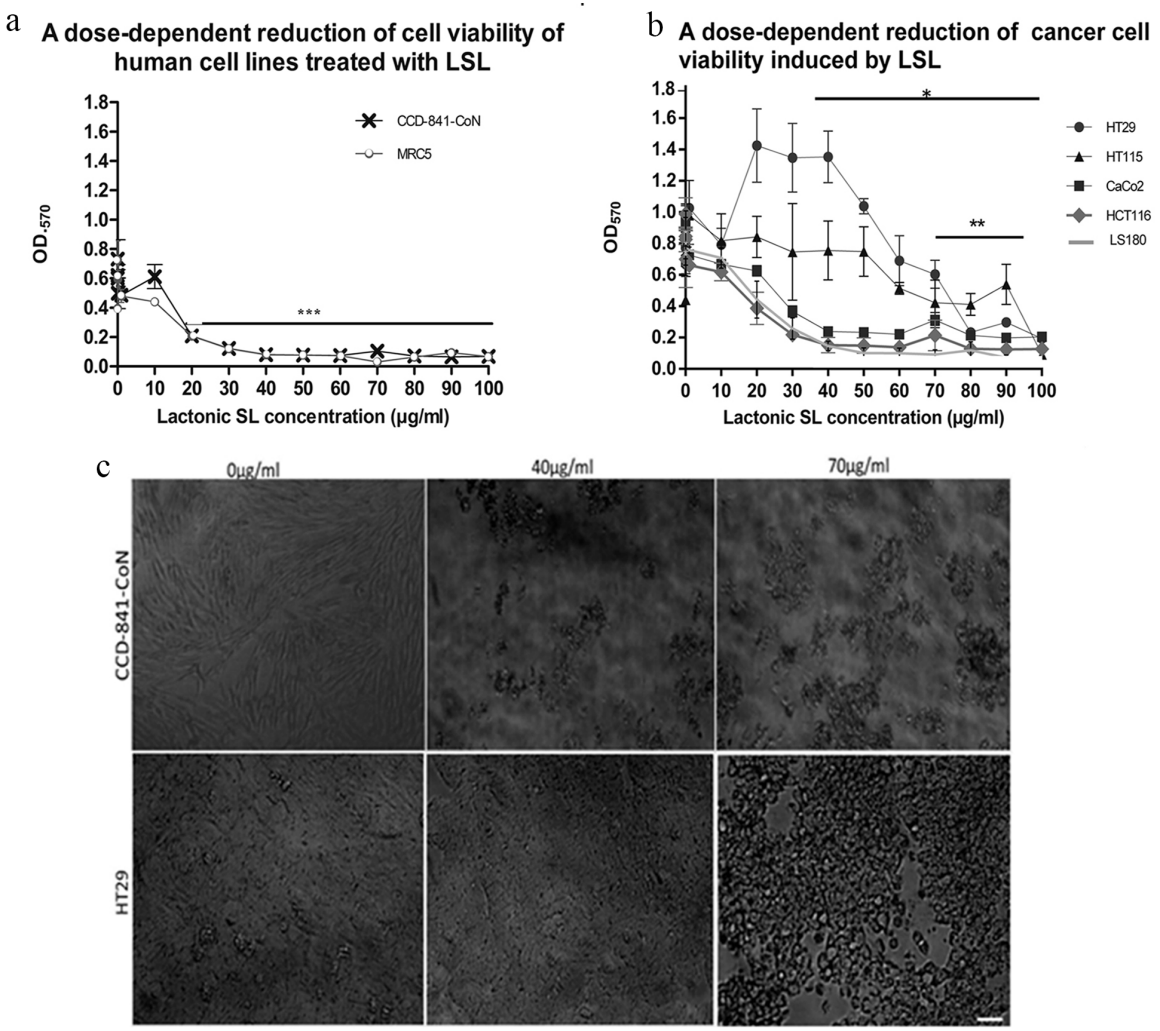
LSL administration causes a dose-dependent effect on cell viability and morphology. (a) Following 24h of treatment with LSL we observed a significant reduction in O.D.570 values at doses between 20–100μg/ml (p<0.0001) in the control cell lines CCD-841-CoN and MRC5. (b) There was a significant reduction in O.D.570 values at doses between 30–100μg/ml in HCT116, Caco2 and LS180 (p < 0.05) and at doses above 60μg/ml inn HT29 and HT115 cells (p < 0.001). Graphs show a representative data set from three independent experimental replicates. Values indicate mean ± SEM (n = 6). Statistical significance was assessed one-way ANOVA (*p < 0.05; **p < 0.001; ***p < 0.0001). (c) Light micrographs from CCD-841-CoN colonic epithelial cells (top) treated with vehicle-only control (left) where cells show a typical bipolar morphology. After treatment with 40μg/ml (central) or 70μg/ml (right) LSL the remaining adherent cells are rounded and there are large regions devoid of cells. In HT29 cultures treated with vehicle-only (bottom right) the cells are densely packed with a cobblestone-like morphology. Following treatment with 40μg/ml LSL (middle), there are no discernable changes in cell morphology, however treatment with 70μg/ml LSL (right) leads to cell rounding and conspicuous cell free areas. Scale bar = 50μm for all images.

In culture, LSL concentrations above 20μg/ml resulted in reduced viability of both colonic epithelial (CCD-841-CoN) and lung fibroblast (MRC5) cell lines (Fig 3a; p<0.0001), in addition to Caco2, HCT116 and LS180 colorectal tumor cell lines (Fig 3b; p < 0.05). HT29 cells initially appear to increase in viability at doses between 20–40μg/ml; however this phenomenon is not statistically significant from vehicle-only control values (p ˃ 0.05). In both HT29 and HT115 colorectal cancer cell lines a significant decrease in viability was observed at doses exceeding 70μg/ml (p < 0.001; Fig 3b). Microscopic examination of confluent cultures of CCD- 841-CoN cells revealed a bipolar morphology following exposure to vehicle, whereas at doses of 40 and70 μg/ml there were large areas devoid of cells, with remaining adherent cells displaying a shrunken and rounded phenotype (Fig 3c: top). In vehicle-treated cultures, HT29 cells display densely packed, cobblestone-like morphology (Fig 3c: bottom) and there was no obvious change in phenotype at a dose of 40 μg/ml LSL. In HT29 cells exposed to 70μg/ml LSL, the confluent monolayer was disturbed and there were clear signs of cell rounding and cell-free areas indicative of detachment (Fig 3c: bottom). Detached cells were isolated from the supernatant of wells treated with 0, 50 and 100μg/ml LSL to determine if they were alive or dead using propidium iodide and Syto 9 staining. At 50 and 10μg/ml, all cells found in the normal CCD-841-CoN supernatant were dead (S1A Fig p<0.001). In the cancer cell lines, 4% of cells were alive and 96% of detached cells were dead (S1B–S1E Fig p <0.01). At 100μg/ml, all detached colorectal cancer cells were dead (p<0.001).

### LSL induce cell death *in vitro*

**Fig 4:**
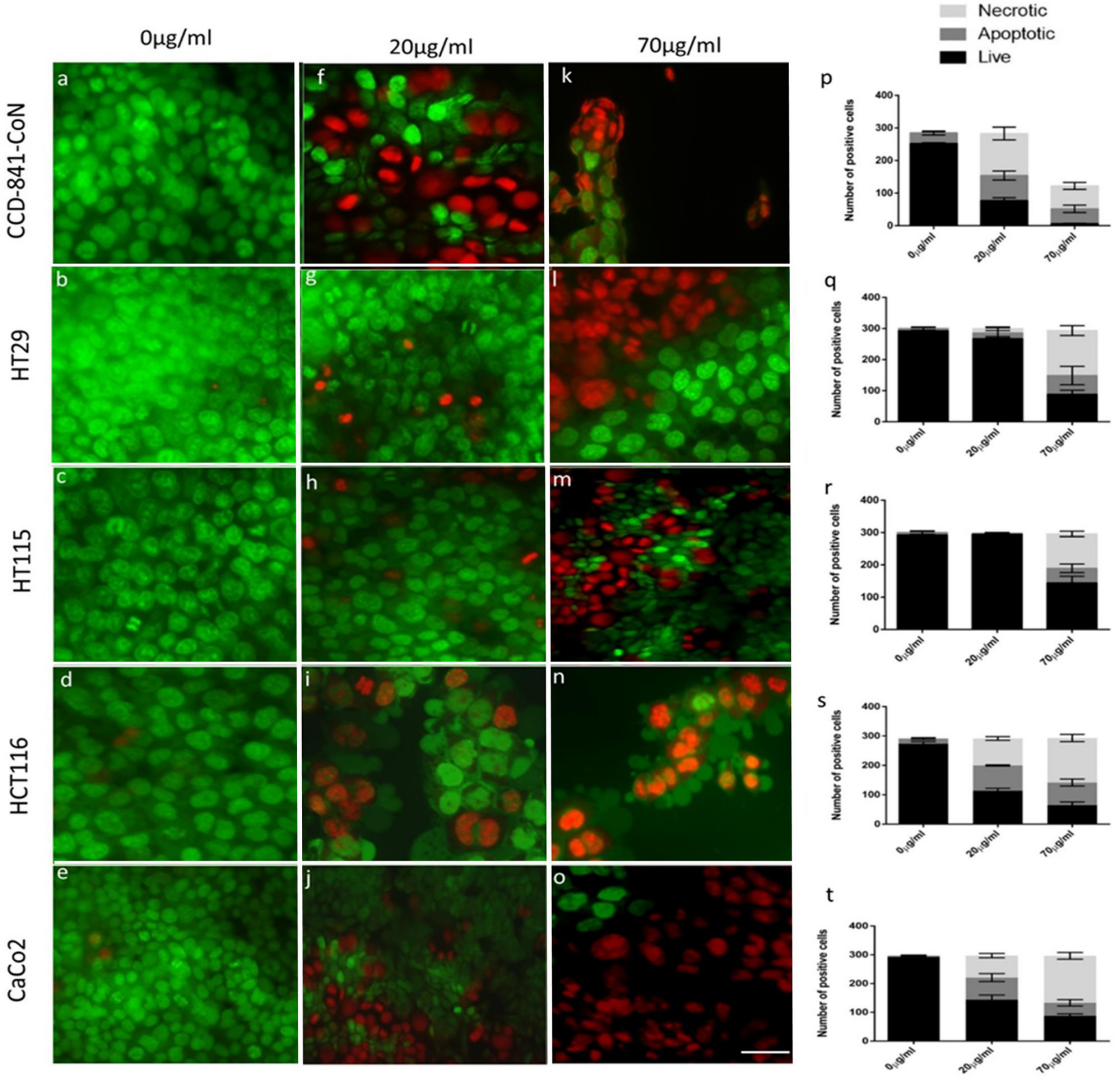
LSL induced both necrosis and apoptosis *in vitro*. Photomicrographs of acridine orange and ethidium bromide stained cultures following treatments with 0 (a-e), 20 (f-j) or 70μg/ml (k-o) LSL and quantification of live, apoptotic or necrotic cells (p-t). The vast number of CCD-841-CoN (a) and cancer cells treated with vehicle control are morphologically viable with a small number showing condensed nuclei (apoptotic). 20μg/ml LSL resulted in necrosis (red/orange clusters) in all cell lines, although CCD-841-CoN (k.p ****p<0.0001) and Caco2 cells (j.o ** p<0.001) were more susceptible. At 70μg/ml, very few adherent CCD-841-CoN cells were observed (k), remaining adhered cells were either necrotic or apoptotic (p). HT29 (l), HT115 (m), HCT116 (n) and Caco2 (o) cells exposed to 70μg/ml LSL all had 50% of cells with morphological features of cells death (q-t) and statistically significant increases in either the numbers of necrotic (*** p<0.0001) or apoptotic cells (**p<0.001) as compared with vehicle only controls.

LSL treatment resulted in a higher proportion of cells undergoing necrosis compared to apoptosis in both normal colonic as well as the four colorectal cancer cell lines we examined (Fig 4). In CCD-841-CoN cultures a dose of 20μg/ml LSL resulted in ~70% cell death (Fig 4f), the majority of which were necrotic (Fig 4p). Both HCT116 (Fig 4i) and Caco2 (Fig 4j) were susceptible to cell death at a dose of 20μg/ml LSL, while HT29 (Fig 4g) and HT115 (Fig 4h) were relatively resistant. In CCD-841-CoN cells exposed to 70μg/ml LSL, the few attached cells available for quantification (Fig 4k) were either necrotic or apoptotic (Fig 4p), whereas in HT29 (Fig 4l), HT115 (Fig 4m), HCT116 (Fig 4n) and Caco2 (Fig 4o) all showed a significant increase in both necrotic (p < 0.0001) as well as apoptotic (p < 0.001) cells (Fig 4q–4t) when compared with vehicle only (Fig 4a–4e).

### Oral administration of LSL to Apc^min+/-^ mice exacerbates tumor development

**Fig 5:**
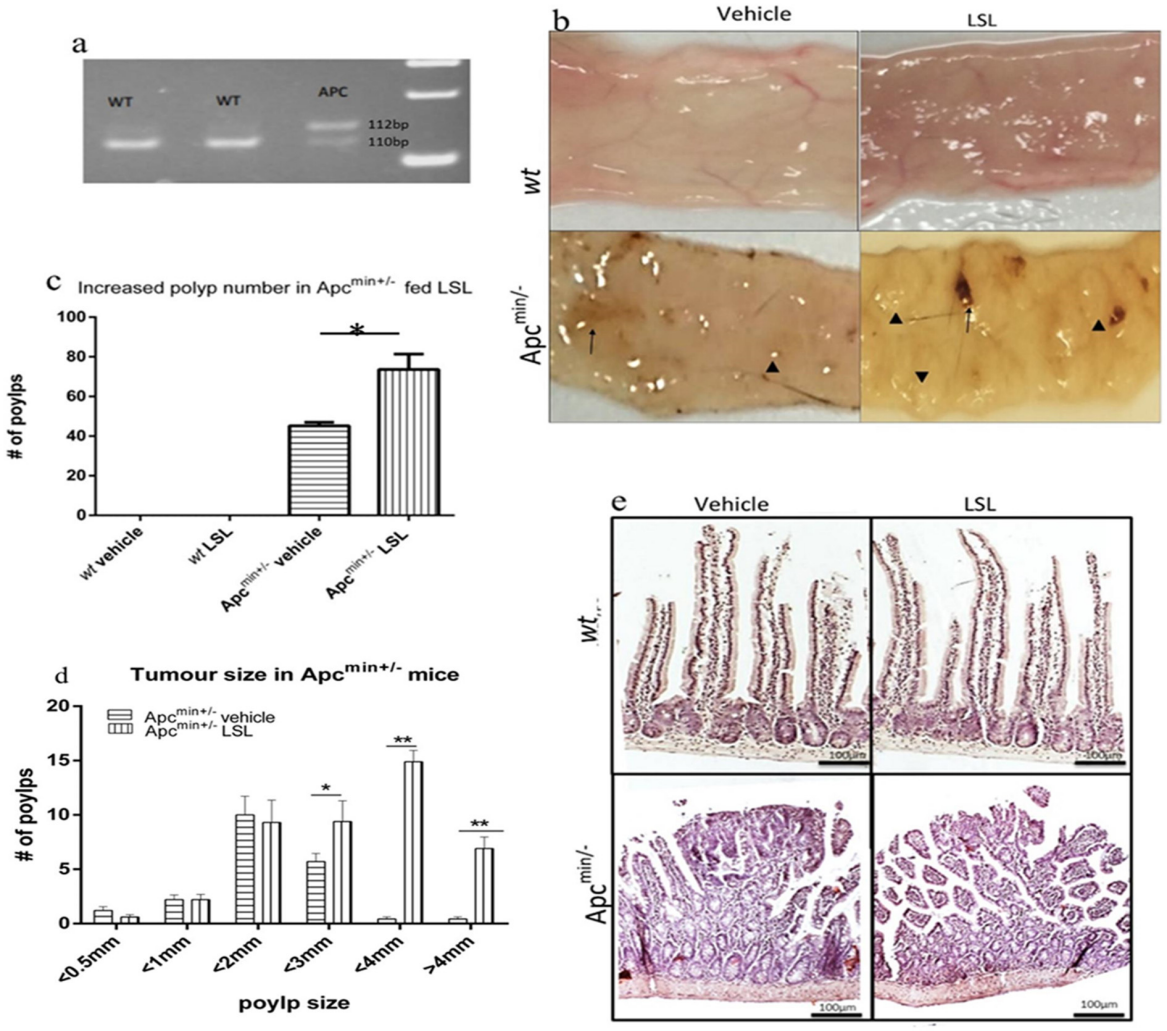
LSL treatment increases tumour number and size in Apc^min+/-^ mice. PCR and restriction enzyme analysis of genomic DNA from wt or Apc^min+/- littermates^ (a); wt band: 111bp, heterozygous Apc^min+/-^ mouse—111 & 123bp. Mice were fed either LSL or vehicle-only every other day for 70 days. Examination of low power images of intestinal segments from wt mice treated with vehicle or 50mg/kg LSL revealed no discernible differences in gross morphology (top). However, in Apc^min+/-^ (bottom), those treated with vehicle-only (left) showed evidence of occult bleeding (arrow) as well as numerous polyps (c arrow heads) with a modal diameter of 2mm (d). In Apc^min+/-^ mice treated with LSL, there was evidence of recent hemorrhage (arrows) as well as significant increases in both polyp number (c;*p <0.01) and size (d;**p < 0.001) compared to vehicle-only controls. Values represent mean ± SEM (n = 10/group). Statistical significance was determined by one-way ANOVA (c) or a student T-tests (d). (e) Histological sections from wt (top) or Apc^min+/-^ (bottom) stained with Haematoxylin and Eosin. Distinct villi in wt treated with vehicle (left) or 50mg/kg LSL are seen. In Apc^min+/-^ littermate mice villous structure is grossly perturbed within polyps in both vehicle-treated mice (bottom left) as well as those treated with 50mg/kg (bottom right).

Genotyping of mice was undertaken following genomic DNA extraction, PCR and subsequent restriction enzyme digestion and electrophoresis; this methodology yielded a single 111bp band for the *wt* allele or dual 111/123 bp alleles (Fig 5a) that are consistent with a heterozygous Apc^min+/-^ mouse. On the basis of genotyping, mice were randomly assigned to either LSL or vehicle-only dosing groups, irrespective of gender. The weights of both *wt* and Apc^min+/-^ mice fed with either vehicle-only control or LSL solutions were not significantly different (25.2g vs 24.9g NS p < 0.1) and there were no differences in water (98.2ml vs 99ml; NS, p > 0.05) or food (180.7g vs 178g; NS p > 0.05) consumption over the duration of the experiment. Dosing of mice for 70 days with LSL also had no effect on the weights of the heart, liver, kidneys or lungs in *wt* mice (data not shown). The gross morphological appearance of unfixed flat mounted ilea from *wt* mice (Fig 5b top) treated with vehicle-only (left) or 50 mg/kg LSL (right) was characterized by a flattened, uniformly smooth mucous epithelium. In vehicle-only treated Apc^min+/-^ mice (Fig 5b; bottom left), there was clear evidence of occult bleeding throughout the ileal segment and numerous polyps (modal diameter 2mm; Fig 5d) compared to *wt* mice. Following treatment with 50mg/kg LSL for 70 days, there is clear evidence of recent bleeding as well as a greater number (vehicle-only = 55.5 ± 3.3 *vs* 50mg/kg LSL = 70.5 ± 7.8; Fig 5c; p < 0.05) of larger diameter (modal size 4mm; Fig 5d; p < 0.001) polyps throughout the ilea compared to the vehicle only treated Apc^min+/-^ mice (p < 0.001). Histological features of sections of *wt* mouse ilea treated with vehicle or 50mg/kg LSL are characterized by evenly spaced, narrow villi with mucoid glands at their base (Fig 5e; top). Sections through Apc^min+/-^ polyps (Fig 5b; bottom) treated with vehicle- only (left) or 50mg/kg LSL (right) reveals a disturbed villous architecture lacking epithelial differentiation.

### LSL treatment specifically increases splenic weight and red pulp proportion in the Apc^min+/-^ mouse

**Fig 6:**
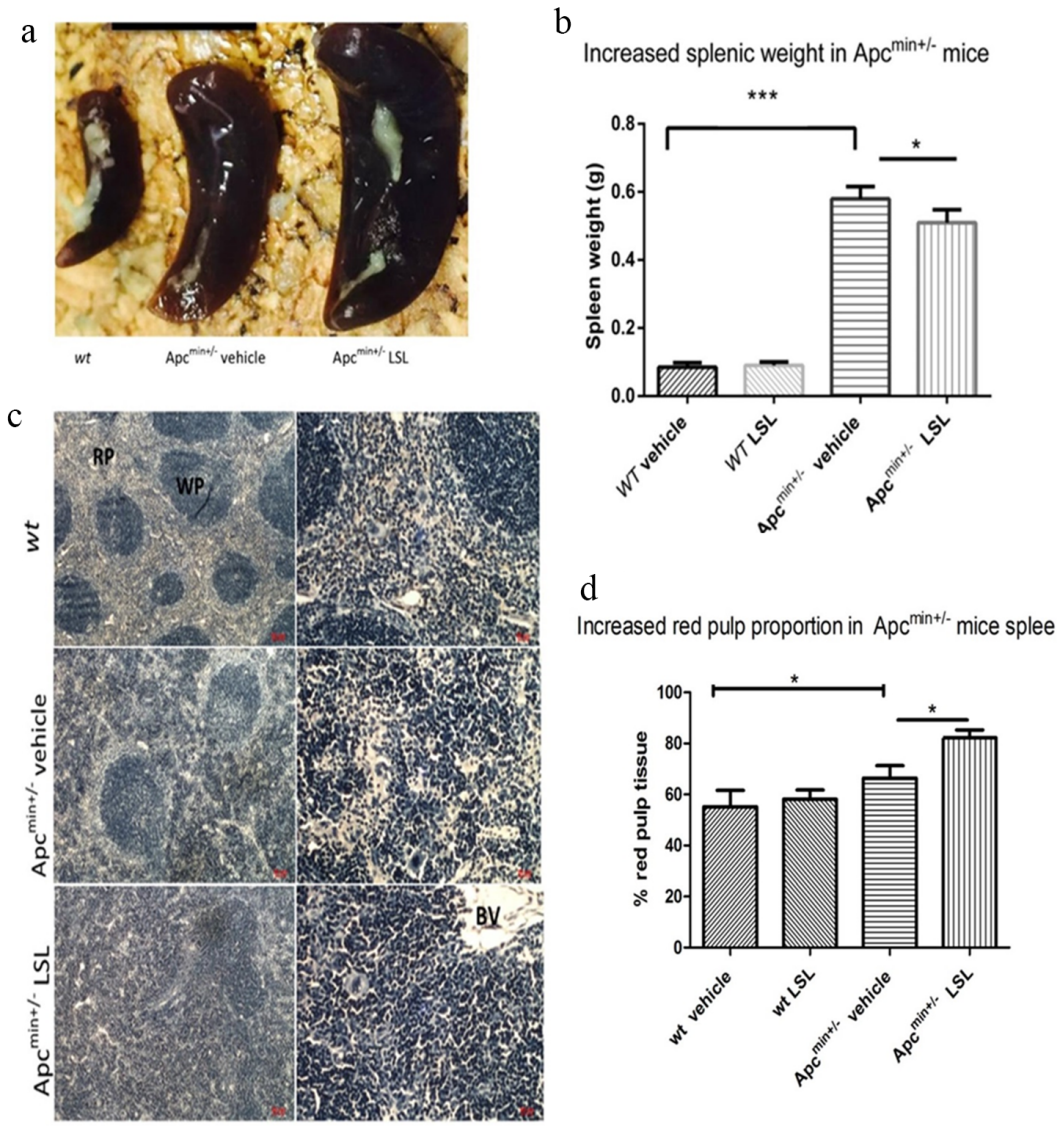
LSL treatment increases spleen size and red pulp area in Apc^min+/-^ mice. a; Photographs of dissected spleen from a wt mouse and Apc^min+/-^ fed with vehicle or LSL. b; Apc^min+/-^ mice had a significantly greater spleen weight compared to wt mice (**** p<0.00001). LSL administration resulted in a significant increase in splenic weights compared to the vehicle control mice (* p<0.01). Splenic histology from wt mice (6c top), Apc^min+/-^ mice fed vehicle control only (6c middle) and Apc^min+/- fed^ LSL (6c bottom). WP = White pulp, RP = Red pulp, BV = Blood vessel. Apc^min+/-^ mice demonstrated altered splenic pathology with increased red pulp regions (hematopoietic rich tissue). LSL fed mice showed a further change in histopathology (*c*.*f* 6c middle vs bottom) with spleens demonstrating a significant increase in red pulp (* p<0.05). Graphs represented of mean ± SEM. Animals per group n = 10. Statistical significance was determined by students T-test.

The weights and gross morphological appearances of heart, lungs, kidneys and liver were not significantly different between either *wt* and Apc^min+/-^ mice or between mice fed either vehicle only or 50mg/kg LSL (NS, p > 0.05; data not shown). Feeding *wt* mice with either vehicle-only or 50mg/kg LSL, also did not affect the wet weights of excised spleen (Fig 6a and 6b; NS, p > 0.05). However, spleens from Apc^min+/-^ mice were both larger (Fig 6a) and heavier (Fig 6b; p < 0.0001) than those from *wt* mice. Administration of 50mg/kg LSL for 70 days to Apc^min+/-^ mice also resulted in an increase in splenic size (Fig 6a) and weight (Fig 6b; p < 0.05). Examination of histological sections from *wt* mouse spleen (Fig 6c; top) revealed conspicuous intensely basophilic areas of white pulp, separated by less dense regions of red pulp in the areas responsible for removal of old or damaged erythrocytes. In Apc^min+/-^ mice the proportion of red pulp was increased compared to *wt* (*c*.*f*. Fig 6c top and middle; p < 0.05). Following treatment with 50mg/kg LSL there was a further increase in red pulp size as compared with vehicle-only controls (*c*.*f*. Fig 6c middle and bottom p < 0.05). Hematocrit values were significantly higher in *wt* than Apc^min+/-^ mice (49.5 ± 0.9 vs 38.1± 1.2; p <0.001). Additionally, feeding Apc^min+/-^ mice with 50 mg/kg LSL for 70 days caused a significant decrease in hematocrit compared to the vehicle-only control (38.1± 1.2 vs 28.2 ± 1.8; p<0.05).

## Discussion

In order to decrease dependency on petrochemical derived surfactants, biosurfactants are increasingly finding use in a variety of applications ranging from industrial and household cleaning reagents through to skin-care products and foodstuffs [8]. The organism with the highest productivity yield of biosurfactants is the pathogenic species *Pseudomonas aeruginosa*, which has made their large scale industrial and health-care use problematic [34]. Modified strains of the yeast *Starmerella bombicola* is a potential commercially viable alternative, as it is non-pathogenic and a high yielding producer of homogenous SL.

In addition to their current commercial uses, SL preparations have previously been reported to have anti-cancer activity based on their ability to reduce the viability of pancreatic [19], lung [21], liver [24] and esophageal cancer cells *in-vitro*. However, these aforementioned studies are difficult to compare, as inter-study variation is significant and the purity as well as homogeneity (proportion of sophorolipid species) is often unreported. Since both purity of SL as well as homogeneity [22] can affect the outcome of biological responses to these molecules, we produced a pure (99% SL) and homogeneous LSL preparation (96% C18:1) for subsequent use in *in vitro* and *in vivo* experiments. SL is formed as a complex mixture with related species differing by the degree of sophorose acetylation as well as fatty acid length and saturation. This species diversity, coupled with various congeners found in crude SL preparations makes separation and purification difficult, demanding and expensive; however it is vital when considering potential pharma-therapeutic uses. In the past number of years, purification of SL has been achieved by the use of thin layer chromatography, HPLC and column chromatography [35]. The majority of studies investigating the anti-cancer potential of SL separate and purify samples with the use of HPLC, MS or NMR [19, 21] producing data showing the exact composition of the SL to be tested, but the information on purity and composition is often omitted. One notable study [16] which used pure and well characterized SL (92% 18:1 LSL) examined their effects on breast carcinoma cells and found a dose-dependent cytotoxic effect. Here we report on a 96% 18:1 LSL preparation that was used throughout our *in vitro* and *in vivo* experiments. We addressed whether a pure preparation of LSL from *Starmerella bombicola* has a differential and/or dose-dependent effect on transformed adherent cells in comparison to “normal” adherent cells; a highly desirable property for potential cancer chemotherapeutics [36]. We assessed five well characterized colorectal cancer cell lines (HT29, HT115, HCT115, LS180 and CaCo2) in addition to adherent, non-transformed colonic epithelium (CCD-841-CoN) and lung fibroblasts (MRC5). LSL had the capability to discriminate in their ability to induce cell death in these cell types; however, they have a more potent effect against “normal” cells at lower doses (10μg/ml). Only a small number of studies have been carried out looking at the anti-cancer activities of SL isolated from *Starmerella bombicola*, such as the breast cancer line MDA-MB-231 (92% C18:1 LSL). A majority of studies have been carried out using SL produced by *Wickerhamiella domercqiae* which was recently identified as *C*. *bombicola* after genome sequencing [37]. These cytotoxicity studies [24], also demonstrate similar potent effect of SL from doses ranging from 40μg/ml– 2mg/ml. The wide range of dose efficacy may be partially explained by the differences in SL species and uncharacterized mixtures. The repeatability and high level of consistency in the data from our in vitro and in vivo studies is consistent with our conclusions on the biological activity of our LSL sample, although we cannot exclude the possibility that the 3.8% ASL found within our SL mixture has a co-incident biological activity.

LSL mixtures had no effect on circulating (non-adherent) blood monocytes, although their comparison with adherent pancreatic tumor cells is spurious. Other non-transformed cell lines examined in the literature include the uncharacterized, and not readily available HL7702 and the ‘Chang’ liver cells [24, 25] believed to derived from normal liver, but later found to be HeLa contaminated [38].

We determined that colorectal cells supplemented with 40–70μg/ml LSL begin to die after 24hr *in vitro*. The predominant type of cell death observed, following ethidium bromide/acridine orange staining, was necrosis. This occurred at doses of 70μg/ml in the cancer cell lines and 20μg/ml in the normal cell lines. Necrosis is a type of unregulated programmed cell death [39], characterized by the disruption of the lipid membrane resulting in the leakage of intracellular proteins, reduction in ATP and cell lysis thus provoking an immune response [40]. SL induced necrosis has been demonstrated in other cell lines, as quantified by LDH release: such as HPAC [16] and the HL-60 leukemic cell line [41].

The induction of necrosis in various cell lines (including those described in this study) likely occurs *via* the intercalation of biosurfactants into the lipid bilayer as has been previously documented [42]. Koley *et al*., 2010 explained that, at a cell-line specific minimal concentration, surfactants integrate into the cell lipid membrane, resulting in carbon chain structural rearrangement. High doses induce tension at the interfacial region of the bilayer, resulting in phospholipid dehydration which affects lipid stability, cellular adhesion and function [43]. This ultimately results in cell death [44], which is evident in studies of SL induced membrane disruption in sperm [18].

Studies investigating the therapeutic potential of SL *in vivo* are limited with the exception of sepsis models. SL mixtures reduce mortality in rats with experimentally induced sepsis via cecum puncture. However in comparison to the natural mixtures—LSL has caused an unexpected increase in the mortality rate in the septic rats at the same dose [20].

The Apc^min+/-^ mouse is a popular animal model to investigate the correlation between food, genetics and chemotherapeutic in the development of intestinal adenomatous neoplasms (polyps). These mice have a life span of <150 days due to secondary consequences of the disease (extensive bleeding of colonic polyps accompanied by anemia) thus making it an ideal and quick model to study the effects of compounds [28, 29, 45, 46]. Oral administration was chosen as the ideal route of administration, in contrast to a traumatic abdominal injury, as it allows the LSL to have direct access to the gut epithelium and polyps to exert their biological effect. Considering the ability of SL mixtures to reduce cancer cell viability, it is surprising that we could find no reports of the *in vivo* use of these SL in established pre-clinical models of cancer development. As our studies indicated an effect of LSL on a range of colorectal cancer cell lines at dosages that would be tolerated for oral administration, we hypothesized that long-term administration would slow progression of colorectal tumors in the Apc^min+/-^ mouse model.

The results show that orally administered purified LSL did not decrease polyp development, but instead caused the exacerbated growth of adenomatous polyps in the intestinal pre-cancerous Apc^min+/-^ mouse model. LSL treatment also increased the size (volume) of the polyps which is currently used as an indicator of tumor burden [47].

The use of other markers is useful in determining disease progression. Apcmi^min+/-^ naturally present with an enlarged spleen and reduced hematocrit as a result of colorectal bleeding [45, 46]. Our study showed that LSL administration resulted in a further increase in spleen size and reduced hematocrit compared to the vehicle control mice. The increase in spleen size may be due to the role it has in clearing out dead and defective erythrocytes [48]. The effect has been documented with other drug administration in mice such as benzo(a)pyrene, an immunomodulatory drug [49].

In conclusion, LSL do not discriminate in their ability to induce cell death between transformed and normal cell lines, as well as increasing progression in the pre-clinical Apc^min+/-^ mouse model. This study is therefore instructive in urging caution concerning the interpretation of *in vitro* studies examining potential anti-tumor effects of purified preparations of LSL and SL in general.

## Supporting Information

S1 Fig LSL treatment results in the detachment of dead cells. Quantification of live or dead cells using propidium iodide and Syto 9 staining on cells extracted from the supernatant of cultures treated with 0, 50 or 100μg/ml of LSL. Following treatment with 50μg/ml LSL, CCD-84l CoN (a), all detached cells in the supernatant were dead (*** p <0.001). At the same concentration, a small number of colorectal cancer cells (b-e) were alive (4%) while the remainder were dead (96% *p< 0.01). In CCD-84l-CoN control cultures and colorectal cancer cells exposed to 100μg/ml LSL, 100% of cells counted in the supernatant were dead (*** p <0.0001). Graphs representative of mean ± SEM. Significance was calculated using a student’s t-test and one-way ANOVA.
(TIF)
