# Supplementary figures and images for: Lactonic Sophorolipids Increase Tumor Burden in Apcmin+/- Mice
Source: PLoS One. 2016 Jun 6;11(6):e0156845. doi: 10.1371/journal.pone.0156845 (PMC4894592; doi:10.1371/journal.pone.0156845)

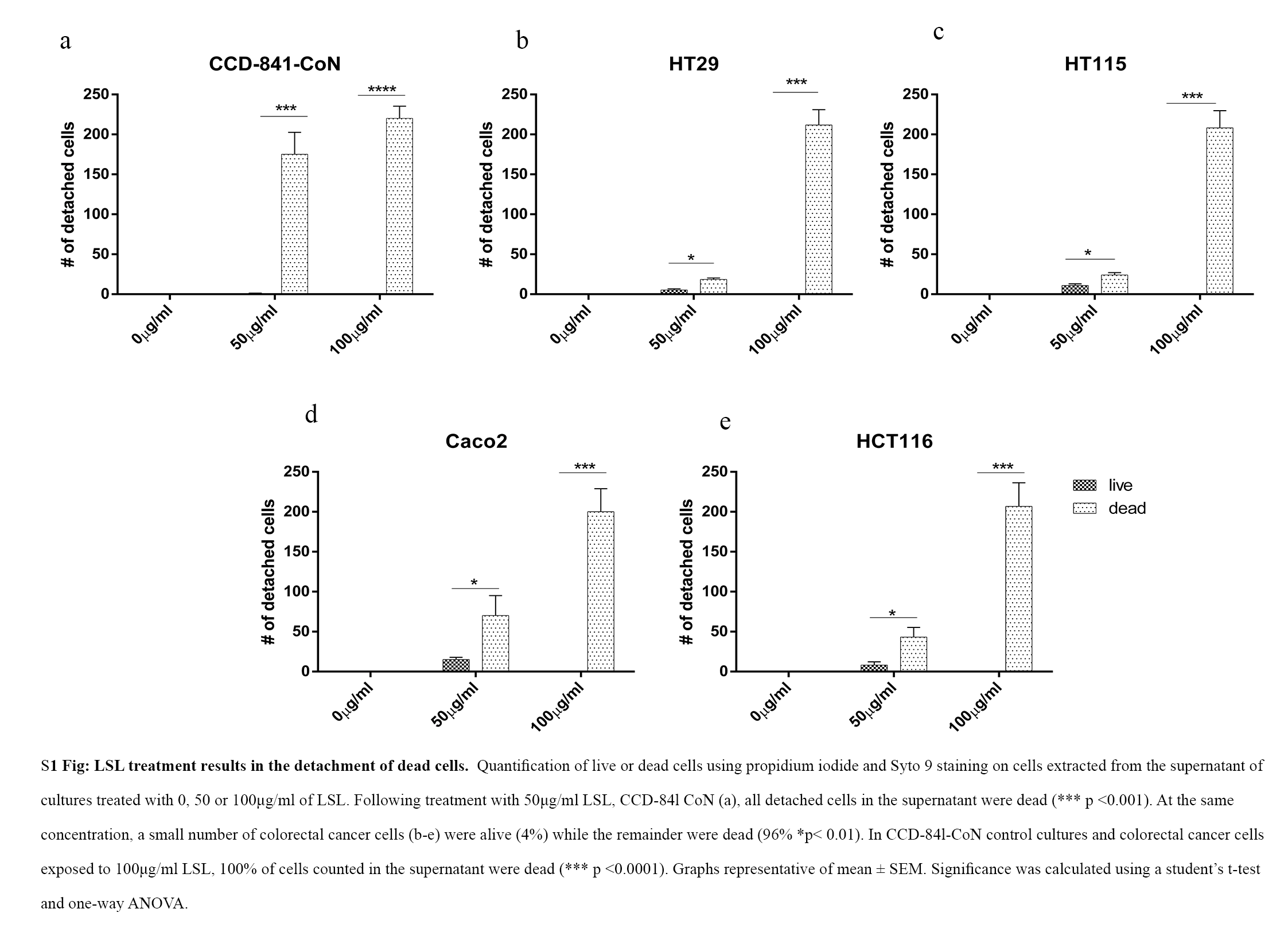

Supplement: S1 Fig — Quantification of live or dead cells using propidium iodide and Syto 9 staining on cells extracted from the supernatant of cultures treated with 0, 50 or 100μg/ml of LSL. Following treatment with 50μg/ml LSL, CCD-84l CoN (a), all detached cells in the supernatant were dead (*** p <0.001). At the same concentration, a small number of colorectal cancer cells (b-e) were alive (4%) while the remainder were dead (96% *p< 0.01). In CCD-84l-CoN control cultures and colorectal cancer cells exposed to 100μg/ml LSL, 100% of cells counted in the supernatant were dead (*** p <0.0001). Graphs representative of mean ± SEM. Significance was calculated using a student’s t-test and one-way ANOVA. (TIF) [file pone.0156845.s001.tif]
